# Immunomodulatory Activity of Low Molecular-Weight Peptides from *Nibea japonica* in RAW264.7 Cells *via* NF-κB Pathway

**DOI:** 10.3390/md17070404

**Published:** 2019-07-08

**Authors:** Zhuangwei Zhang, Xuyang Hu, Lin Lin, Guofang Ding, Fangmiao Yu

**Affiliations:** 1 Zhejiang Provincial Engineering Technology Research Center of Marine Biomedical Products, School of Food and Pharmacy, Zhejiang Ocean University, Zhoushan 316022, China; 2 ZhouShan Academy of Agriculture and Forestry Sciences, Zhoushan 316022, China

**Keywords:** *Nibea japonica*, protein hydrolysates, Low molecular-weight peptide, RAW264.7 cell, immunomodulatory

## Abstract

In this study, a low molecular-weight (Mw) peptide named NJP (<1 kDa), was purified from a protein hydrolysate of *Nibea japonica* by ultrafiltration, and its immunomodulatory effect on RAW264.7 cells was evaluated. The lactate dehydrogenase (LDH) and MTT assays were performed to explore the cytotoxicity of NJP. The results showed that NJP promoted cell proliferation and had no significant toxic effects on RAW264.7 cells. Moreover, the cells formed multiple pseudopodia indicating that they were in activated state. Further tests showed that NJP significantly promoted phagocytic capacity, and the secretion of proinflammatory cytokines tumor necrosis factor-α (TNF-α), interleukin-6 (IL-6), and interleukin-1β (IL-1β). It also increased the synthesis of nitric oxide (NO) by upregulating inducible nitric oxide synthase (iNOS) protein level. Flow cytometry revealed that NJP promoted cell cycle progression and increased the percentage of cells in G0/G1 phase. NJP promoted IκBα degradation, p65 and nuclear factor (NF)-κB activation and translocation by up-regulating IKKα/β protein expression. In conclusion, these results indicated that NJP exerts immunomodulatory effects on RAW264.7 cells through the NF-κB signaling pathway. Therefore, NJP can be incorporated in the production of functional foods or nutraceuticals.

## 1. Introduction

Bioactive peptides (BAP) are a class of peptides that physiologically regulate cellular activities and consist of 2–20 amino acids. BAP has attracted much attention due to their strong biological activity, few side effects, high food safety, easy absorption, and high specificity [1,2]. Protease enzymatic hydrolysis of holoprotein molecules is commonly used to produce bioactive peptides. The hydrolysates are then fractionated by ultrafiltration to obtain low molecular-weight (Mw) peptides and screened for various biological activities [3]. In particular, low Mw peptides have higher bioavailability and bioactivity than protein molecules due to the digestion of larger proteins in the human gastrointestinal tract [4,5]. Hence, the use of low Mw enzymatic food-derived bioactive peptides to develop functional foods or nutraceutical has become an effective value addition approach for agricultural products. Previous studies have shown that protein hydrolysates derived from various marine organisms possess many bioactivities including immunomodulatory [6,7], anti-inflammatory [8,9], antioxidant [10,11], anticancer [12,13], and antimicrobial [14,15,16] effects. Some marine bioactive peptides have been approved for clinical phase assessment or introduced in the commercial market as pharmaceutical products and functional foods [17].

The immune system regulates various physiological processes of the human body. Therefore, improving the body’s immunity and enhancing immune function is crucial to preventing disease occurrence and restoring health [18]. Macrophages are important for the body to exert immune function. Activated macrophages can directly or indirectly participate in the immune response of the immune system. Activated macrophages can eliminate antigenic foreign bodies, senescent cells and cell debris by phagocytosis, and further secrete reactive oxygen species, reactive nitrogen species and pro-inflammatory factors such as TNF-α and interleukins to participate in the immune response [19]. However, most immunomodulatory drugs are not suitable for therapeutic or prophylactic use for chronic diseases because they are generally toxic and have side effects [20]. Although dietary therapy requires a large dose, people are more inclined to replace drugs with nutritional interventions such as eating functional foods in their daily routine [21]. Several immunomodulatory hydrolysates derived from food proteins can be used as functional foods or natural active products. Yang et al. [22] prepared marine oligopeptide preparation (MOP) from the by-product of minced meat processed from Chum Salmon (*Oncorhynchus keta*). They found that MOP can enhance cellular immunity, humoral immunity and NK cell function in mice. Enzymatic protein hydrolysates from green microalga *Chlorella vulgaris* can stimulate the mononuclear phagocytosis system of undernourished mice, and also restore the humoral and cell-mediated immune function [23]. Duarte et al. [24] prepared fish protein concentrate (FPC) from *Merluccius productus* by fermentation. The phagocytic activity of peritoneal macrophages in FPC-fed mice was enhanced, and the levels of some pro-inflammatory cytokines such as IFN-γ and TNF-α were increased.

Giant Croaker (*Nibea japonica*) is a marine commercial fish found in the Indian-West Pacific region, and usually inhabits the lower niches of a river, the estuary, the reef, and the beach area [25]. Members of our laboratory previously described the extraction and characterization of collagen from the skin of *Nibea japonica* [26,27]. To our knowledge, few studies have studied the immunomodulatory potential of low Mw peptides in protein hydrolysates from *Nibea japonica* flesh. In this study, the protein hydrolysate of *Nibea japonica* was prepared by papain. Then, the Mw characteristics of the hydrolyzates were determined using ion exchange chromatography, followed by ultrafiltration to obtain peptide fragments with different Mw distributions. The immunomodulatory activity of the peptide NJP with the highest proliferative rate on RAW264.7 murine macrophages was further evaluated. The results of this study are expected to reveal more opportunities for developing Giant Croaker flesh products for chronic diseases.

## 2. Results and Discussion

### 2.1. Determination of Mw Distribution

The Mw distribution determines the biological and functional properties of protein hydrolysates [28]. To investigate the Mw distribution of the protein hydrolysates from *Nibea japonica*, the retention time (Rt) was plotted against the logarithm of the Mw (lg_Mw_) [29].

$$\mathrm{lg}\mathrm{Mw}=-0.2753R_{t}+7.3148$$

**Figure 1:**
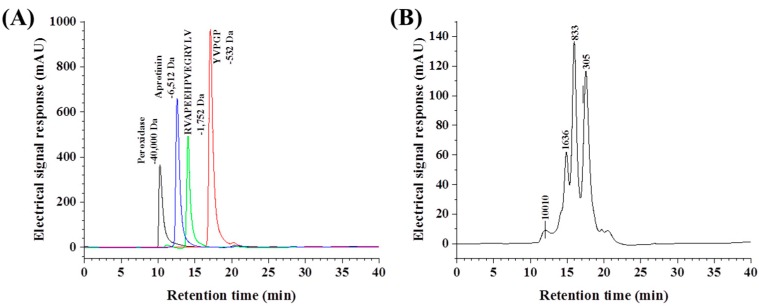
(**A**) High-performance liquid chromatograms of standard molecular weight samples. Standard molecular weight samples: Peroxidase-40,000 Da, Aprotinin-6,500 Da, Pentadecapeptide RVAPEEHPVEGRYLV-1,750 Da, and Pentapeptide YVPGP-530 Da); (**B**) the molecular weight distribution of NJPs; (HPLC: Agilent 1260 Infinity II HPLC, Column: TSK gel G2000 SWXL (7.8 × 300 mm, 5 µm), Mobile phase: CH_3_CN/H_2_O/TFA = 45:55:0.1, Flow rate: 0.5 mL/min.

**Figure 2:**
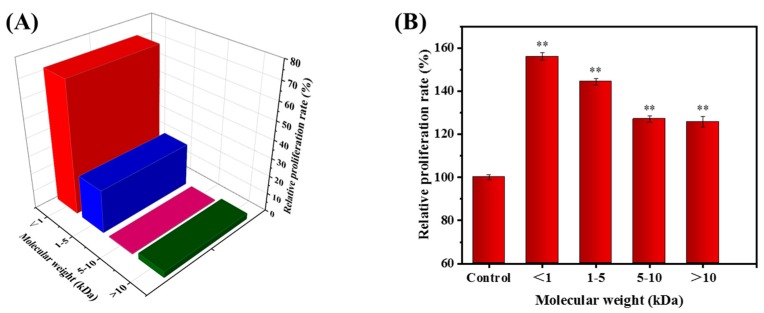
Profile of molecular weight distribution of NJPs (**A**) and its effect on the activity of RAW264.7 cells (**B**). * *P* < 0.05 and ** *P* < 0.01 vs. Control group.

The calibration equation of measuring coefficient (R^2^) was 0.9652, indicating a good linear relationship for the 100,000–100 Da compound (Figure 1A). Figure 1B showed the elution profile of protein hydrolysates from *Nibea japonica* (NJPs) under the same chromatographic conditions. Then, the Mw of the NJPs was obtained by the substitution of the retention time in the calibration equation [30]. The Mw distribution of NJPs was expressed in Figure 2A, which was mainly consisted of peptides with lower Mw (72.056% of the fraction below 1 kDa), and the other peptide fractions with higher Mw in the range of 1–5 kDa (24.075%). The exogenous peptides with small Mw are more easily absorbed by the human body in a complete form [5]. In addition, peptide fraction with low Mw structures has high solubility due to the presence of polar amino acid units and stronger hydrogen bonding with water molecules [28,31]. This indicated that the low Mw fraction of *Nibea japonica* protein hydrolysate can be commercially exploited as a food ingredient. Therefore, we isolated and characterized the functional properties of the active fractions of the protein hydrolysates.

### 2.2. Fractionation of the NJP from NJPs

According to the principle of mechanical interception, the enzymatic hydrolyzates from *Nibea japonica* flash were separated by ultrafiltration membranes with different Mw cutoffs into four fractions with different Mw distributions (<1 kDa, 1–5 kDa, 5–10 kDa, and >10 kDa). Then, the effect of each fraction on the proliferation of macrophages was determined. As shown in Figure 2B, the peptide fraction below 1 kDa produced the highest proliferation of RAW264.7 cells (56.01%, *P* < 0.001 vs. control) compared to other ultrafiltration fractions. Similar studies have shown that low Mw fractions purified from protein hydrolysates have higher biological activities such as antioxidant [32,33,34], antihypertensive [35,36] and immunomodulatory [37,38]. The results also showed that the biological activity of the hydrolysate was dependent on the Mw distribution. Thus, the fraction with Mw of <1 kDa was selected for subsequent activity evaluation and named as NJP.

### 2.3. Cytotoxicity of NJP on RAW264.7 Cells

**Figure 3:**
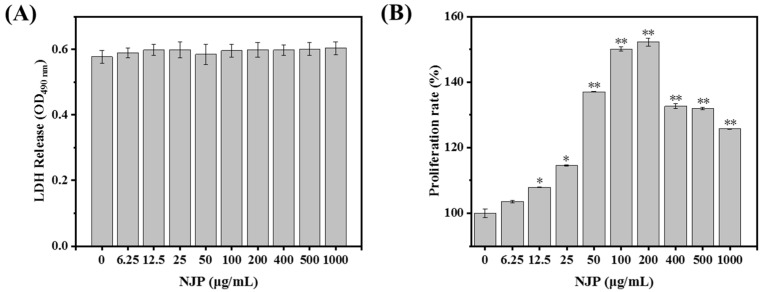
Effects of different concentrations of NJP-treatment for 24 h on lactate dehydrogenase (LDH) release (**A**) and cell proliferation rate (**B**) in RAW264.7 cells. * *P* < 0.05 and ** *P* < 0.01 vs. Control group.

Lactate dehydrogenase (LDH) is an oxidoreductase that exists in the cytoplasm of all normal cells. In normal cells, cytoplasmic LDH cannot pass through the cell membrane. However, when cell membrane permeability is increased due to damage, LDH with stable enzyme activity is released from the cells [39]. The activity of LDH in the supernatant of a cell culture medium can be used to determine the degree of cell damage, and hence the cell membrane integrity in the quantitative detection of cytotoxicity [40]. To investigate whether NJP has toxicity effects on RAW264.7 cells, we measured the release of LDH in the presence or absence of NJP (Figure 3A). Data showed that NJP treatment had no significant effect on LDH release from RAW264.7 cells at a wide range of concentrations 6.25~1000 μg/mL (*P* > 0.05, vs. 0 μg/mL NJP-treated cells). Subsequently, we evaluated the cytotoxicity of NJP by measuring the proliferation of RAW264.7 cells *via* MTT viability assay.

### 2.4. Effect of NJP on RAW264.7 Cells Viability

Before any compound is used for clinical treatment, it should be known to have no adverse effects on cellular metabolism. Macrophages are monocyte-derived phagocytic cells, which play an important role in innate immune defense and acquired immune responses. Therefore, the proliferative activity of macrophages can be used as an indicator of the involvement of immune activators in cytotoxicity [41,42]. The results of the MTT assay demonstrated that NJP treatment had a significant effect on RAW264.7 macrophage viability at the indicated concentrations of 12.5~1000 μg/mL (Figure 3B). It also increased the relative proliferation rate of RAW264.7 cells was significantly increased in a dose-dependent manner at different concentrations (6.25, 12.5, 25, 50, 100, and 200 μg/mL). However, the viability of RAW264.7 cells slightly decreased when the NJP concentration increased between 400 and 1000 μg/mL), on the other hand, the viability of RAW264.7 cells was not slightly influenced by NJP treatments at the indicated concentrations.

### 2.5. Effect of NJP on RAW264.7 Cells’ Morphology

**Figure 4:**
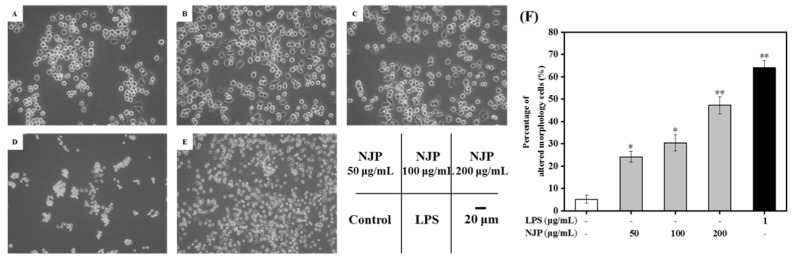
Effect of NJP on morphology of RAW264.7 cells treated with 50 (**A**), 100 (**B**), 200 (**C**), and 0 (**D**) μg/mL of NJP and 1 μg/mL of LPS (**E**), respectively (×200). The red arrow represents the existence of the pseudopod. (**F**) The percentage of cells with differentiated morphology in 6-cells culture plate. * *P* < 0.05 and ** *P* < 0.01 vs. Control group.

The morphology of macrophages changes with the cell activity, function, and environment. When stimulated with LPS or other signaling factors, the morphology of RAW264.7 cells was altered [43]. In this study, morphological changes of RAW264.7 cells treated with NJP or LPS were investigated under an inverted microscope. The macrophages of the Control group were round with normal morphology. But treatment with NJP and LPS induced morphological changes in RAW264.7 cells. The cells stimulated by LPS exhibited polygonal and irregular shapes accompanied by formation of many pseudopods, which increased the contact area with external substances and enhanced the phagocytic uptake and adhesion of cells. After NJP treatment, the cell body was enlarged, and it displayed a mixture of a circular shape and irregular shape, with pseudopod extension. The pseudopod gradually elongated as the concentration of NJP increased (Figure 4). Similar to the study by Hang et al. [44], these morphological changes reflected the activation of macrophages. Furthermore, the relative number of RAW264.7 cells with differentiated morphology revealed a concentration-dependent effect with NJP (Figure 4).

### 2.6. Effect of NJP on Phagocytosis

**Figure 5:**
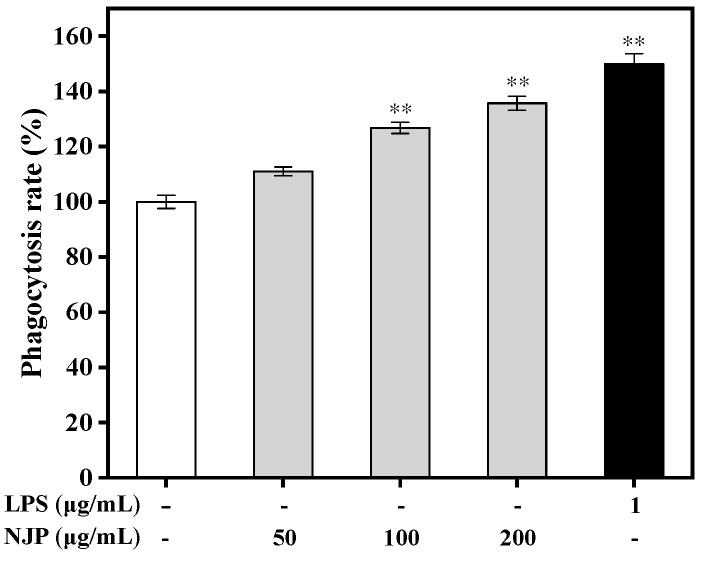
Effect of NJP on phagocytosis of RAW264.7 cells by neutral red internalization model. * *P* < 0.05 and ** *P* < 0.01 vs. Control group.

Phagocytosis influences immune response and the clearance of apoptotic cells or cell debris [45]. In this process, macrophages internalize particulate matter such as microorganisms. The neutral red dye internalization model is commonly used to evaluate the phagocytic function of RAW264.7 cells. Here, the phagocytic activity was determined after treatment with LPS or different concentrations of NJP using the neutral red assay (Figure 5). Compared with the control group, the phagocytosis rate of neutral red was increased following NJP treatment in a dose-dependent manner. RAW264.7 cells treated with 100 and 200 μg/mL NJP had higher internalization of neutral red than Control group cells (*P* < 0.01). In addition, the phagocytosis rate of RAW264.7 cells after treatment with 200 μg/mL NJP (135.74%) was increased, at a rate equivalent with treatment of 1 μg/mL LPS (149.92%). Similarly, the increased dose of ADPs-1a and ADPS-3a in *A. dahurica* were bound up with the increase of the phagocytic capacity of RAW264.7 cells to neutral red [46]. These data indicated that NJP improved the phagocytic capacity and non-specific immunity of RAW264.7 cells within a specific concentration range.

### 2.7. Effect of NJP on NO Production and iNOS Expression

**Figure 6:**
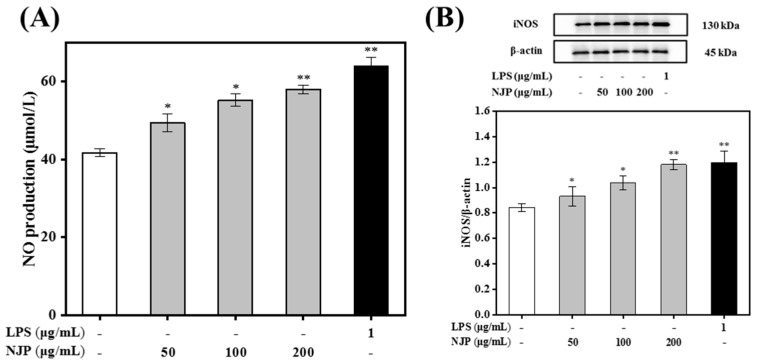
Effect of the NJP on NO production (**A**) and the expression of inducible nitric oxide synthase (iNOS) protein (**B**) in RAW264.7 cells treated with different concentrations of NJP (0, 50, 100, and 200 μg/mL) and 1 μg/mL of LPS for 24 h. * *P* < 0.05 and ** *P* < 0.01 vs. Control group.

NO is an inorganic gaseous free radical which plays a pivotal role in the non-specific immune system, by activating macrophages [47]. The synthesis of NO is mainly mediated by iNOS, a subtype of the nitric oxide synthase (NOS) family isoenzymes. The mRNA and protein expression of iNOS in macrophages is initiated by stimulation of cytokines such as interferon-γ (IFN-γ), TNF-α, and lipopolysaccharide (LPS). iNOS catalyzes the synthesis of NO and L-citrulline from L-arginine through an NADPH- and oxygen-dependent mechanism. The production of NO in NJP-treated macrophages was measured by NO assay kit (Nitrate reductase method). As shown in Figure 6 the production of NO changed in a dose-dependent pattern following NJP treatment. The amount of NO secreted from RAW264.7 cells treated with increasing concentrations of NJP (50, 100, and 200 μg/mL) was remarkably higher than that of the Control group. Furthermore, endogenous levels of total iNOS protein were detected by electrophoretic transfer. There was significant difference in iNOS protein level between 50, 100 μg/mL-NJP treatment and the control group. In addition, NJP significantly upregulated the protein levels of iNOS at 200 μg/mL-NJP (*P* < 0.01). These results indicated that NJP-treated RAW264.7 cells expressed more iNOS protein and produced more NO, which is in agreement with previous studies on *Coix glutelin* protein hydrolysates (≤3 kDa), a protein isolated from *Panax quinquefolius* L [45] and polysaccharides from *Spirogyra neglecta* (Hassall) Kützing [48].

### 2.8. Effect of NJP on TNF-α, IL-6, and IL-1β

**Figure 7:**
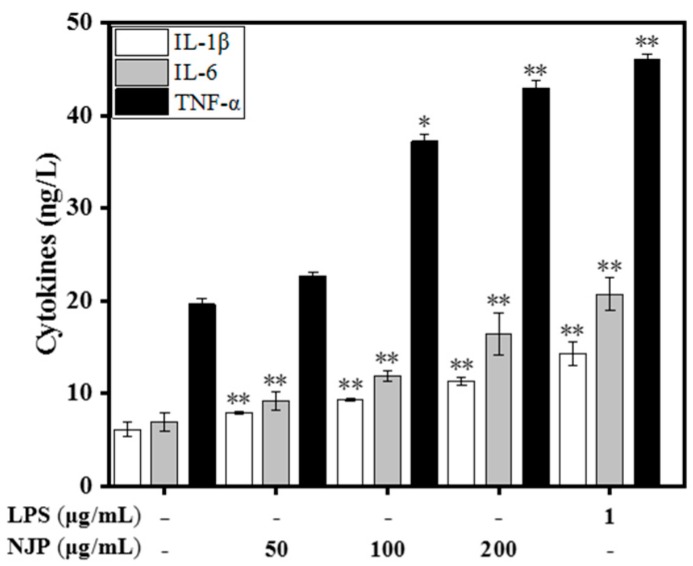
Effect of NJP on the secretion of proinflammatory cytokines IL-1β, IL-6, and TNF-α in RAW264.7 cells. * *P* < 0.05 and ** *P* < 0.01 vs. Control group.

Pro-inflammatory cytokines are a class of low Mw soluble polypeptides secreted by activated macrophages that have different effects on the function of native and acquired immune system [44]. The effects of lymphokines and endotoxins on macrophages are mediated by TNF-α, which is a key modulator of immune diseases. Macrophages synthesize IL-1β in response to inflammatory signals, which further trigger the production of other inflammatory cytokines such as IL-6 in target cells. This synergistically promotes and amplifies the inflammatory response leading to tissue damage [49,50]. Thus, TNF-α, IL-1β, and IL-6 were used to evaluate the immunomodulatory and anti-inflammatory effects of the compound. The levels of TNF-α in the supernatant increased significantly, which was positively correlated with the dose of NJP treatment. Similarly, the production of IL-1β and IL-6 increased following NJP treatment in a dose-dependent manner. Our data suggested that NJP effectively increased the production of pro-inflammatory cytokines in RAW264.7 cells (Figure 7). These findings are consistent with the results of morphological observation, neutral red phagocytosis, and NO production, showing a good dose-dependent response.

### 2.9. Effect of NJP Cell-Cycle Distribution of RAW264.7 Cells

**Figure 8:**
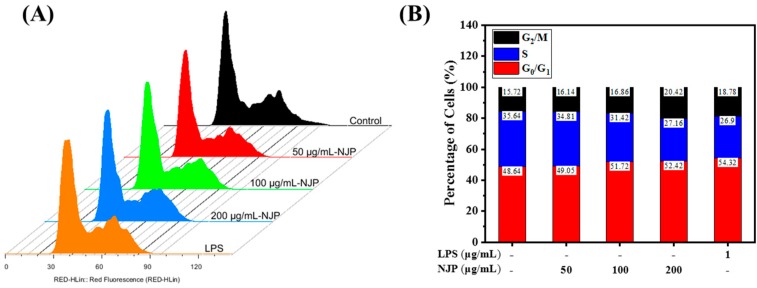
(**A**) Effects of NJP on the cell cycle progression of RAW264.7 cells; (**B**) percentages of G0/G1, S, and G2/M phase in the RAW264.7 cells.

Cells undergo an orderly and repetitive cycle of division; from the end of one division to the end of the next division (G0/G1→S→G2→M), which is called the cell cycle. Different DNA contents at different cell cycle stages result in variation of nucleic acid binding to fluorescent dye PI, which is analyzed by flow cytometry [51]. To further characterize the effect of NJP on cell proliferation, the cell cycle distribution of PI-stained cells was assessed (Figure 8). The percentage of cells in G0/G1 phase was markedly increased following NJP treatment relative to untreated Control cells (*P* < 0.05). However, the cell ratios at S and G2/M stages decreased in a dose-dependent manner. Flow cytometry indicated that NJP promoted entry of more RAW264.7 cells into G0/G1 phase but decreased the number of cells in S and G2/M phase, thereby accelerating the cell cycle and promoting cell proliferation [52].

### 2.10. Effect of NJP on NF-κB Signal Pathway in RAW264.7 Cells

**Figure 9:**
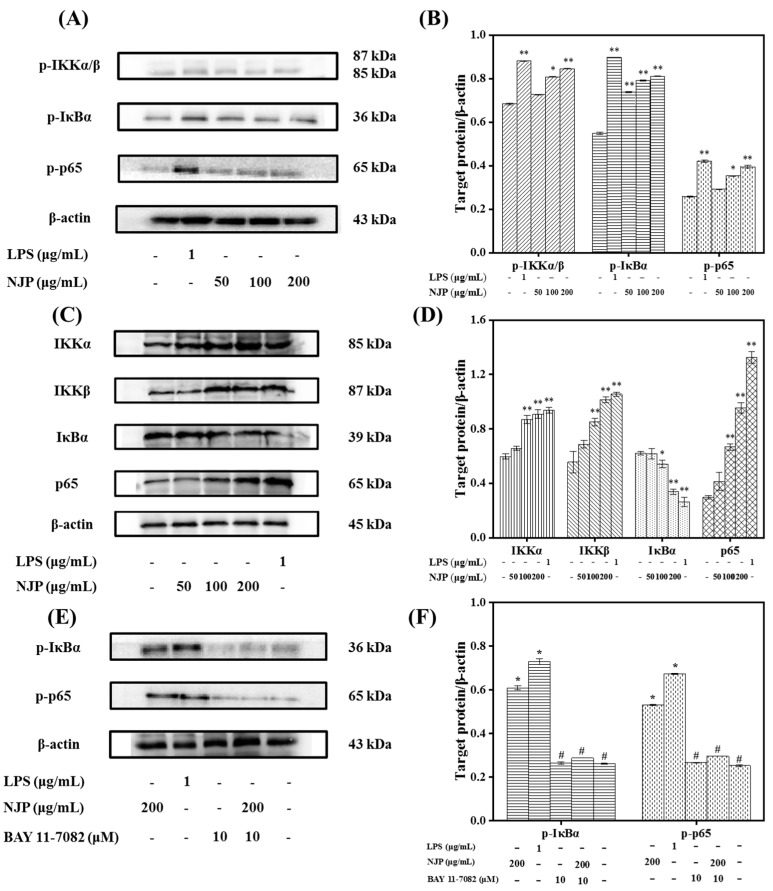
Effects of NJP on the expression of NF-κB pathway-related proteins in RAW264.7 cells. (**A**) Western blot analysis of p-IKKα/β, p-IκBα, and p-p65 induced by NJP in RAW264.7 cells. (**B**) The protein levels of p-IKKα/β, p-IκBα, and p-p65 induced by NJP in RAW264.7 cells. (**C**) Western blot analysis of IKKα, IKKβ, IκBα, and NF-κB p65 induced by NJP in RAW264.7 cells. (**D**) The protein levels of IKKα, IKKβ, IκBα, and NF-κB p65 induced by NJP in RAW264.7 cells. In (**A**), (**C**), and (**E**), β-actin was used as the loading control. In (**B**), (**D**), and (**F**), * *P* < 0.05 and ** *P* < 0.01 vs. Control group, # *P* < 0.05 vs. 200 μg/mL NJP.

**Figure 10:**
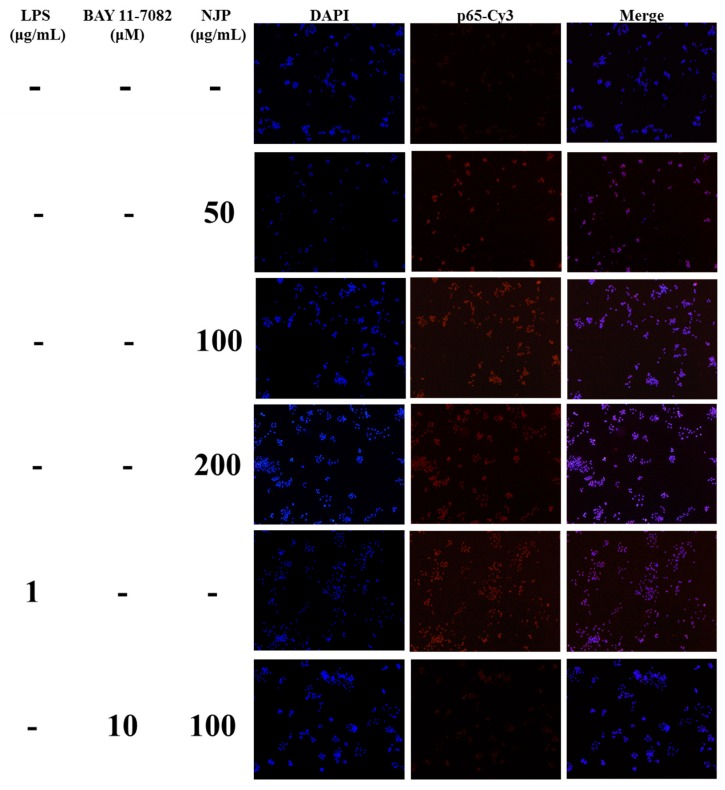
Effects of NJP on the translocation of NF-κB p65 in RAW264.7 cells by immunofluorescence images.

The IκB kinase (IKK) protein complex of the NF-κB signaling pathway activates the phosphorylation and ubiquitination degradation pathway of IκBα protein, which triggers the release of NF-κB dimer [42]. The activated NF-κB dimer enters the nucleus and binds to genes with NF-κB-binding site, which initiates the transcription and expression of inflammatory mediators and pro-inflammatory cytokines. The NF-κB protein family is an indispensable nuclear transcription factor in the regulation of immune responses, inflammatory responses and oxidative stress [53]. Therefore, to n the role of NF-κB signaling pathway in NJP-promoting macrophage activation, the activation and translocation of related proteins were assessed by western blot and immunofluorescence. As shown in Figure 9, the levels of IKKα, β and phorylated re up-regulated, while the level of IκBα decreased with the increased level of phosphor-IκBα. Afterwards, NF-κB p65 and phospho-p65 in cytoplasmic protein (Figure 9), as well as NF-κB p65 in nuclear protein were increased significantly (Figure 10) in RAW264.7 cells after treated with 50, 100, 200 μg/mL NJP. Moreover, specific inhibitor BAY 11-7082 was used to confirm the dependence of NJP on the NF-κB signaling pathway. With the pretreatment of BAY 11-7082, the phosphorylation of IκB was effectively inhibited in NJP-induced RAW264.7 cells. And the degradation of IκBα and nuclear transportation of NF-κB p65 were inhibited (Figure 9 and Figure 10).

These results showed that NJP significantly promoted the dissociation of NF-κB p65 and IκBa by activating IKKα and IKKβ. Furthermore, the release and nuclear transfer of NF-κB p65 were up-regulated, and the expression of inflammation-related genes was increased (like the increased production of pro-inflammatory cytokines in NJP-treated RAW264.7 cells). This may be due to the fact that IκB is degraded by the phosphorylation-ubiquitin-proteasome pathway once IKK cascade is activated. This releases the final NF-κB dimer into the nucleus where it regulates in the expression of relevant immune response genes [54,55,56]. It indicated that NJP exerts immunomodulatory effects by activating NF-κB signaling pathway.

## 3. Materials and Methods

### 3.1. Materials and Reagents

*Nibea japonica* was identified by Prof. Zhao Shenglong from the Department of Marine Biology, Zhejiang Ocean University. The flesh of *Nibea japonica* was harvested and stored at −20 °C in our laboratory.

Murine mononuclear macrophage leukemia RAW264.7 cells were purchased from the National Infrastructure of Cell Line Resource Shanghai Branch (Shanghai, China) and frozen in liquid nitrogen until use. Fetal bovine serum (FBS) was purchased from Zhejiang Tianhang Biotechnology Co., Ltd. (Huzhou, Zhejiang, China). Dulbecco’s modified eagle medium (DMEM) liquid medium and Penicillin-Streptomycin Solution were obtained from Biosharp Biotechnology Co., Ltd. (Hefei, China).

The papain used for scientific research was purchased from YaTaiHengXin Biotechnology Co., Ltd. (Beijing, China). Dimethylsulfoxide (DMSO) and LPS from *Escherichia coli O55: B5* were purchased from Merck KGaA (Darmstadt, Germany). The ultra-pure methylthiazolyldiphenyl-tetrazolium bromide (MTT), Neutral Red cell proliferation and cytotoxicity test kit, lactate dehydrogenase (LDH) cytotoxicity assay kit, NF-κB activation, nuclear translocation assay kit and ECL chemiluminescence reagent were purchased from Beyotime Biotechnology (Shanghai, China). Bicinchoninic acid (BCA) protein assay kit and Radio immunoprecipitation assay (RIPA) Lysis Buffer were obtained from Solarbio Science & Technology Co., Ltd. (Beijing, China). Assay kits for TNF-α, Interleukin -1β and 6, NO were obtained from Jiancheng Bioengineering Institute (Nanjing, China). Antibodies against β-actin (cat. no.13E5), IκBα (cat. no. 44D4), NF-κB p65 (cat. no. D14E12), IKKα (cat. No. AF0198), and IKKβ (cat. No. AI137) were purchased from Cell Signaling Technology (Boston, MA, USA). Moreover, Phospho-IKKα/β (Ser176/180), Phospho-IKB alpha (Ser32), Phospho-NF-κB p65(Ser536) rabbit monoclonal antibodies and NF-κB inhibitor BAY 11-7082 were provided by Beyotime Biotechnology. All reagents used were of analytical grade.

### 3.2. Preparation of NJPs

The flesh of *Nibea japonica* was homogenized (JJ-2 Kinematica, Bilon Instrument Co., Ltd., Shanghai, China), mixed with 95% ethanol at a ratio of 1:6 (*w*/*v*, g/mL), and stirred at 50 °C for 1 h to degrease three times. The degreased mixture was then centrifuged at 12,000 rpm for 15 min using a CF16RN high-speed micro centrifuge (Himac, Tokyo, Japan). The precipitate was washed with pure water until there was no ethanol odor and then stored at –20 °C after lyophilization (Christ Alpha 1–4 LD plus Laboratory freeze dryer, Marin Christ, Germany). The protein hydrolysate of *Nibea japonica* flesh was prepared using papain under the conditions reported by Hu et al. [57]. A specific amount of the defatted flesh was mixed with water at 1:11 (*w*/*v*, g/mL), and papain with an enzyme concentration of 2000 U/g was added. After heating to 59 °C for 5.4 h, the pH of the reaction mixture was maintained at 6.0 by addition of a small amount of 0.5 M Sodium hydroxide or 0.5 M hydrochloric acid intermittently using a DG150 pH detection-balance system (Lohand Biological Technology Co., Ltd., Hangzhou, China). Afterwards, the enzymatic reaction was terminated by keeping the reactants in boiling water bath for 10 min, and then the precipitate was removed by centrifugation at 12,000 rpm for 10 min. Finally, the supernatant was collected and freeze-dried for further study.

### 3.3. Mw Distribution

The Mw distribution of NJPs was analyzed using Agilent 1260 Infinity II HPLC (Agilent Technologies, Inc., CA, USA) with a TSK gel G2000 SW_XL_ analytical column (7.8 × 300 mm, 5 µm, TOSOH, Tokyo, Japan) at 220 nm, and the mobile phase of acetonitrile (CAS#: 75-05-8) /H_2_O/TFA (CAS#: 76-05-1): (45:55:0.1) at a flow rate of 0.5 mL/min [58]. The standard samples consisted of peroxidase (40,000 Da), aprotinin (6500 Da), Pentadecapeptide RVAPEEHPVEGRYLV (1750 Da), and Pentapeptide YVPGP (530 Da), which were loaded into the analytical column in turns. The standard curve of R_t_-lg_Mw_ was then plotted. The sample solution was filtered (using 0.22 μm microspore film) and loaded into the column. Finally, the Mw distribution of the NJP was calculated according to the calibration curve equation of Mw.

### 3.4. Fractionation of the NJP by Ultrafiltration

The peptide fraction with the highest immune activity was purified by GM-18 Roll film separation system (Bona Biotechnology Co., Ltd., Jinan, China) with 10, 5, and 1 kDa Mw cut off (MWCO) membranes (Nanostone Water, Inc., MN, USA). Fractions with MW > 10 kDa, MW 5–10 kDa, MW 1–5 kDa, and MW < 1 kDa were collected, lyophilized, and then evaluated for immunomodulatory activity [59].

### 3.5. Analysis of the Immunomodulatory Activity of the Peptide on RAW264.7 Cells

#### 3.5.1. Culture of Macrophage RAW264.7 Cells

RAW264.7 cells were cultured in vitro at 37 °C in a 5% CO_2_ atmosphere (Forma 3111 CO_2_ incubator, Thermo Forma, Waltham, MA, USA). The DMEM supplemented with 10% FBS and containing 100 U/mL penicillin and 100 μg/mL streptomycin was used as the complete medium.

#### 3.5.2. Cytotoxic Test

Destruction of cell membrane integrity leads to the release of cytoplasmic enzymes. For instance, lactate dehydrogenase (LDH) released following cytotoxicity is an important indicator of cell membrane integrity [60]. Measurement of the cytotoxic effects of NJP to RAW264.7 cells was performed using the LDH cytotoxicity assay kit following the supplier’s specifications. Briefly, when RAW264.7 cells reached a confluence of approximately 80%, they were prepared as single cell suspension with complete medium, and then seeded in 96-well cell culture plates (200 μL /well) at a density of 1 × 10^5^ cells/mL and cultured for 12 h. Afterwards, the culture medium was replaced with different concentrations of NJP (0, 6.25, 12.5, 25, 50, 100, 200, 400, 500, and 1000 µg/mL) dissolved in a complete medium. The cells cultured with a complete medium without the peptide served as the control groups. Six parallel wells were prepared in each group and incubated for additional 24 h. Next, the 96-well plates were centrifuged in a multiwell plate centrifuge (Min2596, Zhuhai Hema Medical Instrument Co., Ltd., Zhuhai, China) at 400 rpm for 5 min. The supernatant was subsequently aspirated and 150 μL of working fluid (LDH release reagent: PBS = 1:10 *v*/*v*) was added and incubated for 1 h. The 96-well plates were centrifuged again in a multi-well plate centrifuge (400 rpm, 5 min), and 120 μl of the supernatant of each well was aspirated and added to a new cell culture plate. Immediately, the optical density (OD) value of each well was read at a wavelength of 490 nm with an automatic microplate reader (SpectraMa, Molecular Devices Co., San Jose, CA, USA).

#### 3.5.3. Cell Viability Assay

The MTT assay reported by Karnjanapratum [61] was used to assess the effect of NJP on RAW264.7 proliferation. The RAW264.7 cells with a confluence of approximately 80% were seeded on a cell culture plate with suitable size (1 × 10^5^ cells/mL, 200 μL/well) and monolayer cultured for 12 h. The RAW264.7 cells were treated with NJP as described in Section 3.5.2 and the cells cultured with only complete medium were considered as blank groups. 200 μL of MTT (5 mg/mL with PBS) was added to each well and cultured in an incubator for 4 h in the dark. Thereafter, the supernatant was totally removed and 150 μL of formazan solution-DMSO were added to each well. The 96-well plates were protected from light and vibrated on Mini Shaker (MH-2, Haimen Kylin-Bell Lab Instruments, Haimen, China) for 10 min at 25 °C to fully dissolve the blue-violet formazan crystals. The detection wavelength of the automatic microplate reader was set to 570 nm, and the OD of each well was measured. The viability of RAW264.7 cells after treatment with NJP was reflected by the proliferation rate, and the OD value of each group was calculated by the following equation:

Proliferation rate (%) = [(OD_Treated_ − OD_Blank_)/(OD_control_ − OD_Blank_)] × 100

#### 3.5.4. Morphological Evaluation

The RAW264.7 cells grown to a confluence of approximately 80% were seeded on TC-treated flat-bottomed cell culture plate with 6 wells (1 × 10^5^ cells/mL, 2 mL). After overnight incubation, the cells were treated with different concentrations of NJP (50, 100, and 200 μg/mL). LPS-treatment (1 μg/mL, dissolved in complete medium) and complete medium were used as the positive and control groups, respectively. All RAW264.7 cells treatments were performed in a humidified incubator with 5% CO_2_ atmosphere at 37 °C for 24 h. Finally, an optical microscope (Biological microscope CX31, Olympus, Japan) was used to evaluate and photograph the morphology of RAW264.7 cells. The cells with altered morphology was quantified as the method by Kocbach Bølling et al. [62].

#### 3.5.5. Phagocytosis of Neutral Red

The phagocytosis of Neutral Red was investigated according to the procedures described by Fang [63] with slight modifications. We seeded 200 μL of single cell suspension (1 × 10^5^ RAW264.7 cells/mL) in 96-well plates and cultured it overnight. The cells in the 96-well plate were divided into 5 treatment groups: Control, Positive, and different concentrations of NJP treatment group, with 6 parallel wells in each group. The culture medium was discarded after the incubation, and the cells were separately treated with a complete medium, LPS (1 μg/mL) or NJP of the same concentration as described in Section 3.5.4 for 24 h. After removal of the supernatant, each well was purged twice with PBS buffer and 20 μL of neutral red staining solution and 200 μL of the complete medium were added. After incubation for 2 h, the supernatant was removed and cells were washed with PBS to remove excess neutral red. 200 μL of neutral red lysate (ethanol/acetic acid 1:1) was added to each well, after which the plate was shaken at 25 °C for 10 min. The automatic microplate reader was used to measure the OD of each well at a wavelength of 540 nm and the phagocytic rate was calculated by the following equation:

Phagocytosis rate (%) = (OD_Treated_ − OD_Blank_/OD_Control_ − OD_Blank_) × 100

#### 3.5.6. Measurement of NO Level and related Inflammatory Cytokines

The RAW264.7 cells were treated with NJP as described in Section 3.5.5, and the negative control and positive groups were included. After 24 h of treatment, the supernatant was collected from the cell culture plates and used to measure NO levels by the NO assay kit [64]. The secretion of inflammatory cytokines TNF-α, IL-1β and IL-6 was also measured by the corresponding enzyme-linked immunosorbent assay (ELISA) kits according to the manuals provided by the Jiancheng Institute of Bioengineering Institute (Nanjing, China) [44,65].

#### 3.5.7. Cell Cycle Analysis

To characterize the effect of NJP on RAW264.7 cells in detail, flow cytometry was used to examine different cell cycle stages following the operation steps described by Li et al. [66] with slight modifications. Briefly, 2 mL suspension of RAW264.7 cells in the exponential growth phase were added into 6-well flat-bottom plates at a density of 1 × 10^5^ cells/mL per well. After 24 h of adherent growth, the treatment procedure described in Section 3.5.4 was performed and cells were incubated for 24 h. The cells were washed with pre-cooled PBS buffer and harvested by centrifugation (800 rpm, 5 min). They were then fixed overnight at 4 °C in 75% ethanol. The ethanol was removed by centrifugation and the cell pellet was washed with pre-chilled PBS. The cells were resuspended in 200 μl of pre-chilled PBS buffer. Next, 20 μL of Rnase A solution was added and immersed in a 37 °C water bath for 30 min. After filtration through a 400 mesh screen, 400 μL of propidium iodide (PI) was added in the dark, mixed gently and incubated at 4 °C for 30 min. Finally, the cell cycle curve was established based on the flow cytometric excitation wavelength of 488 nm (Becton Dickinson, NJ, USA). Analysis of cellular DNA content analysis and cell cycle distribution status were performed using FlowJo (Treestar Software, Ashland, OR, USA).

#### 3.5.8. Immunofluorescence Staining

RAW264.7 cells grown on coverslips in 6-well culture plates were treated with 0, 50, 100, and 200 μg/mL of NJP and 1 μg/mL of LPS for 24 h. According to the manufacturer’s instructions, the immunofluorescent staining kit was used to detect the activation and nuclear translocation of NF-κB. In brief, cells were washed three times with PBS and fixed with 4% paraformaldehyde, and then incubated with the immunostaining blocking buffer for 1 h to reduce non-specific binding. Next, cells were incubated overnight with the specific primary antibody against the p65 subunit of NF-κB at 4 °C. Washing was performed again and the Cy3-conjugated secondary antibody was incubated for 1 h at room temperature. The cells were then stained with 4’, 6-diamidino-2-phenylindole (DAPI) solution for 5 min at room temperature. Finally, the RAW264.7 cells on the coverslips were preserved in anti-fluorescence quench-seal and observed by a fluorescence microscope (Axio Imager A2, Carl Zeiss, Germany). Cy3-conjugated NF-κB p65 protein and DAPI-stained nuclei emit red and blue fluorescence respectively. And the multicolor images with purple fluorescence were created after red and blue images were overlapped.

#### 3.5.9. Western Blot Analysis

Western blotting was performed as described by Jiang [51] with some modifications to measure the protein levels of NF-κB signaling pathway-related proteins. RAW264.7 cells were seeded in suitable size culture bottles (1 × 10^5^ cells/mL) and treated with different concentrations of NJP (0, 50, 100, and 200 µg/mL) or LPS (1 μg/mL) for 24 h. The cells were collected and rapidly lysed in RIPA buffer to extract nuclear and cytoplasmic proteins. Protein concentration was determined using a BCA protein assay kit according to company’s protocol. Denatured protein samples (30 μg) were separated on a 12% SDS-PAGE and the gel was transferred to a polyvinylidene fluoride (PVDF) membrane. Subsequently, the membrane was blocked by incubating with 5% non-fat milk for 1 h, followed by incubation overnight at 4 °C with diluted (1:1000) specific primary antibodies at 4 °C overnight. Finally, the diluted (1:1000) specific secondary antibodies were added and incubated at 25 °C for 1 h to bind to the primary antibodies. The solid support matrix of target protein was visualized by enhanced chemiluminescence using the FluorChem FC3 chemiluminescent gel imaging system (ProteinSimple, Silicon Valley, CA, USA), and the optical output density was quantified using the AlphaView image analysis software (version 3.4.0, ProteinSimple, Silicon Valley, CA, USA). β-Actin was used as an internal control.

### 3.6. Statistical Analysis

All experimental results of this study are presented as the mean ± standard deviation ($\bar{x}$ ± *s*) and data are from at least three independent experiments. A one-way analysis of variance (ANOVA) followed by Tukey’s test was used to compare significant differences between groups with SPSS version 19.0 software (SPSS Inc., Chicago, IL, USA) was used to compare significant differences between groups.

## 4. Conclusions

**Figure 11:**
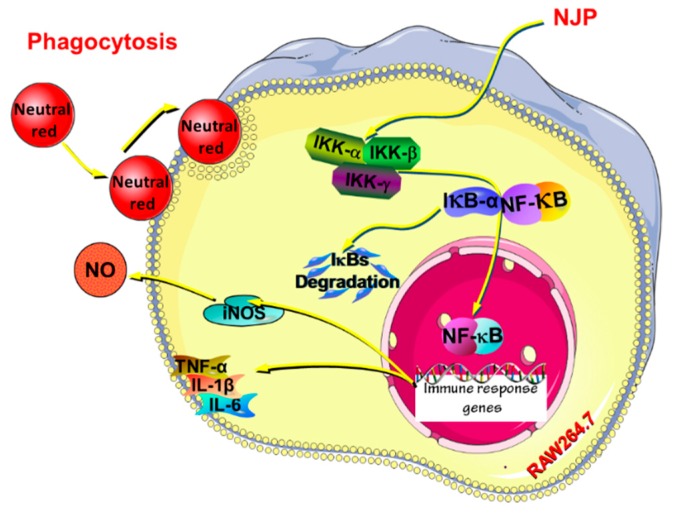
Possible mechanism of NJP exerting immunoregulatory effects in RAW264.7 cells.

In this study, the protein hydrolysate (NJPs) was prepared from *Nibea japonica* flesh using papain. The most abundant low Mw peptide fraction NJP (72.056% of the fraction below 1 kDa) produced the highest proliferation rate of RAW264.7 cells. NJP effectively regulated the proliferation of RAW264.7 cells by increasing the distribution proportion of G0/G1 phase cells and did not exhibit cytotoxicity. Inverted microscopy examination showed that the activation, the phagocytic activity and dendritic morphological changes of RAW264.7 cells increased after NJP treatment in a dose-dependent manner after NJP treatment. NJP upregulated the expression of iNOS protein to promote NO production in RAW264.7 cells. Furthermore, the levels of TNF-α, IL-6, and IL-1β were increased following NJP treatment. Western blotting indicated that NJP increased the protein levels of IKKα and IKKβ, and promoted the degradation of IκB-α as well as the freedom of NF-κB subunit p65 in the NF-κB signaling pathway in RAW264.7 cells (Figure 11). In summary, these findings demonstrate the immunomodulatory effects of NJP in vitro. This compound can be exploited as an immune-promoting ingredient of functional foods and nutritional supplements. Further experiments focusing on functional and metabolic effects in vivo are required.
